# Sex, rurality and socioeconomical status in Spanish centennial population (2017)

**DOI:** 10.18632/aging.203563

**Published:** 2021-09-26

**Authors:** Pedro Fuentes, Sandra Amador, Ana Maria Lucas-Ochoa, Lorena Cuenca-Bermejo, Emiliano Fernández-Villalba, Valeria Raparelli, Colleen Norris, Alexandra Kautzky-Willer, Karolina Kublickiene, Louise Pilote, María Trinidad Herrero, the GOING-FWD Consortium

**Affiliations:** 1 Clinical and Experimental Neuroscience (NiCE), Institute for Aging Research, Biomedical Institute for Bio-Health Research of Murcia (IMIB-Arrixaca), School of Medicine, University of Murcia, Campus Mare Nostrum, Murcia, Spain; 2 Department of Translational Medicine, University of Ferrara, Ferrara, Italy; 3 Faculty of Nursing, University of Alberta, Edmonton, Alberta, Canada; 4 Cardiovascular and Stroke Strategic Clinical Network, Alberta Health Services, Edmonton, Alberta, Canada; 5 Division of Endocrinology and Metabolism, Department of Medicine III, Medical University of Vienna, Vienna, Austria; 6 Department of Renal Medicine, Institution for Clinical Science, Intervention and Technology, Karolinska Institute, Stockholm, Sweden; 7 Research Institute of McGill University Health Centre, Division of Clinical Epidemiology, McGill University, Montreal, Canada

**Keywords:** centenarians, GDP per capita, longevity, rurality, sex

## Abstract

World's population is exponentially aging as people reaching 100 years old has increased. The number of areas with the highest centennial population rates (Blue Zones), are significantly higher. Are there any determinant factors that favor this situation in Spain? The goal of this study was to determine the possible influence of sex, rurality and socioeconomic factors (Gross Domestic Product (GDP)) on the prevalence of the centennial population of the Spanish society. The Spanish register of inhabitants was published in 2017 by the National Statistics Institute.

The analysis was carried out both by Autonomous Communities and by provinces in phases: a first descriptive analysis, followed by an inferential analysis, based on statistical tests (independent T- Student test, Pearson correlation and ANOVA). There were significant interactions between: i) sex and longevity (in favor of the female population); ii) female and rural housing and iii) female, GDP and urban areas.

Feminization was proven in the longevity revolution, but, in general, GDP per Capita was not a significant survival factor on its own. This study was the first step of further analysis related to extreme longevity in Spain, which will include other dependent variables such as state of health and well-being as well as social factors.

## INTRODUCTION

In Spain, the average life expectancy has increased in the last years, having one of the highest life expectancies worldwide with 80.4 years for men and 85.7 for women in 2017 [1]. Life expectancy is higher in women compared to men. In the future, life expectancy in the world will continue to increase being higher in South Korea, some western European countries, and some emerging economies [2]. The place where people live, socioeconomic aspects and the familiar environment (which enriches the immune system with survival aptitudes), are just examples of factors that directly relate with increasing life expectancy and reaching one hundred years old [3–9]; but, why is it a higher density of centenarian people in certain geographic locations? [10] and why women live longer? [11–13].

Despite human genome was described in 2001 [14, 15], the current information is still limited to shed light to many hypothesis and conceptions. Genes that might be involved in the human longevity should be many and varied (involved in different signaling pathways), but none of them seemed to be an individual determinant in longevity and age that a person can reach [16]. It has been stablished that men present a higher heritability influence in survival than women; therefore, female population is more susceptible to environmental influence and, *a priori*, the reason for the greater female survival would be attributed to the environmental variable [17–19]. Malnutrition in all of its forms (obesity and undernutrition) can be translated into dietary risk, which is the leading cause of poor health. In addition, the climate change effects on the health of humans and the natural systems we depend on. This is called The Global Syndemic and it represents the paramount health challenge for humans, the environment and the planet in the 21^st^ century and will affect people’s health [20].

Five regions of the world were described by Dan Buettner in 2004 as those with most centenarians and they were called "Blue Zones", and currently these regions remain the same: Okinawa (Japan), Loma Linda (California), Ikaria (Greece), Nicoya (Costa Rica) and Sardinia (Italy) [10]. Despite of the fact that these regions are widely geographically separated from each other, have heterogeneous customs, different doctrines and very different elements among them, they share series of social agents and principles, classified as the “Nine Powers”: i) people do spontaneous exercise on a daily basis; ii) have reduced stress levels; iii) have a life purpose (goal); iv) follow a control diet - 80% rule or “hara hachi bu”; v) they supervise the healthy components of the diet; vi) people drink wine moderately; vii) the population is believer (even if they are different faiths); viii) individuals have family support; and ix) they feel being part of the right “tribe” [10].

Although there are many studies worldwide [3–7, 21], information about centennial population is scarce in Spain [22–24]. From the few studies that have been previously performed in the centenary population in Spain, we found the following evidences: i) there was feminization of aging in Spain [22–24]; ii) the Autonomous Communities with more centennial people were Asturias, Castilla y León, Galicia, País Vasco, Aragón and Cantabria, while the communities with less centennial people were Canarias, Murcia and Islas Baleares [24]; iii) the areas with the highest density of centenarians in Spain corresponded to regions with a large number of rural areas where the society had dedicated their time for daily work outdoors, such as cattle raising and agriculture; iv) the oldest Spanish rural areas correspond to the Autonomous Communities and provinces of Castilla y León, Asturias, Cantabria, Aragón, Guadalajara and Cuenca [23] and v) the centennial Spanish people stated to have good perceived health, be independent with Activities of Daily Living (ADL) and to own good lifestyle activities such as Mediterranean diet and regular exercise [22].

The hypothesis of this study was that socioeconomic factors in the different Spanish regions (GPD per Capita) as well as the rurality (and sex) could be determinants for the Spanish population longevity (with 2017 data). Then, the main objective of this study was to evaluate the possible impact of sex, socioeconomic factors, and rurality on the prevalence of centennial population (> 95 years) in the Spanish society, through the 2017 continuous population census. The specific objectives were: i) to obtain the geographic differences of the centennial population among the different Autonomous Communities and provinces in Spain; ii) to establish the possible relationship between the prevalence of centenarians with sex variable, geographical factors (rurality), socioeconomic factor based on Gross Domestic Product (GDP) per Capita and iii) the interrelation of these factors.

## RESULTS

All the results obtained in this study are available through dashboards with the Power Bi application. These dashboards are interactive, and they contain two sections. The first deals with the GDP in Spain, in the different Autonomous Communities and provinces. The second shows graphs of the centennial and non-centennial Spanish population, rurality and sex. Filters can be applied to select a specific Autonomous Community or province.

https://app.powerbi.com/view?r=eyJrIjoiYTE0OWVlYTItYmE3Ni00YzA5LWIzMzMtNDlkNjJiZmFlOTBhIiwidCI6IjBhYmE2NTIxLWNlNTItNDRkNy1iMDZjLWM2MDE2ZmYyYzMwYiIsImMiOjh9&pageName=ReportSection

**Figure 1:**
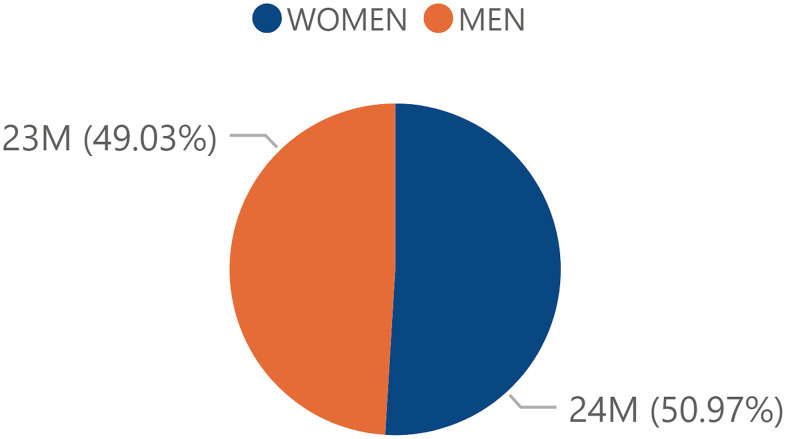
**Percentage of total population by sex.** Percentage of the total Spanish population by sex in 2017 obtained from the INE.

**Figure 2:**
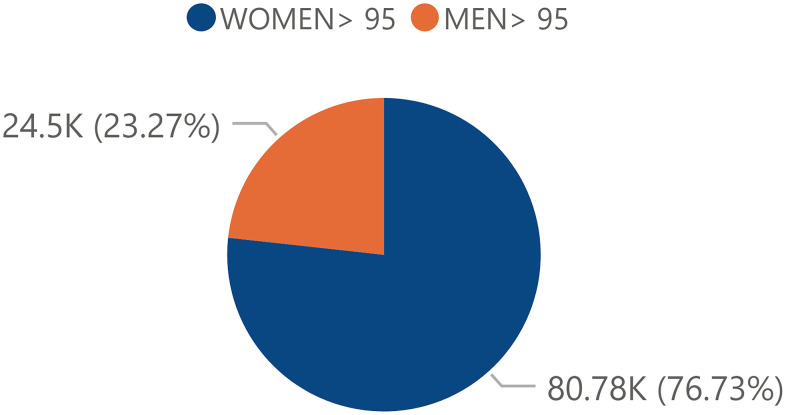
**Percentage of the centennial total population by sex.** Percentage of the total Spanish centennial population by sex in 2017 obtained from the INE.

The distribution of the total male and female general population was similar between men and women with a slightly higher proportion of female population (50.97%) compared to male population (49.03%) (Figure 1). However, the total centennial population in Spain referred a clear trend in terms of its distribution towards the female sex with a percentage of 76.73% and with a male percentage of 23.27% (Figure 2).

Then, at the regional level, there was a slight and non-significant increase shifted towards women. However, a different behavior was described in the communities of Castilla La Mancha and Murcia, and in the Autonomous Cities of Ceuta and Melilla, where the trend was the reverse, towards the male sex (Supplementary Table 1). Likewise, it should be noted that the communities of Asturias, Galicia and Madrid had a lower percentage of males with a clearly positive percentage difference (percentage of female population minus percentage of male). The total centennial population presented the same inclination towards the female sex, but much more pronounced than in the total autonomous population. Cantabria and País Vasco showed more pronounced this trend (Supplementary Table 2).

At the provincial level, the data obtained from the total population were similar to the data obtained at the regional level. In relation to total centennial population, the data by provinces indicate a similar trend. The provinces that showed a higher percentage of female sex were the provinces of the aforementioned Autonomous Communities: Cantabria, Álava, Guipúzcoa and Vizcaya. In second place, they were followed by the provinces of Zaragoza, Palencia, Barcelona and Madrid (Supplementary Table 3).

If the inferential analysis was applied by Student T-Test as a statistic test to evaluate if the differences observed between sexes in the descriptive analysis in the different population groups were statistically significant, at the regional level, the total normalized female population presented an average of 50.67% and a total normalized male population had a mean value of 49.29%. In the total female centennial population, an average of 76.14% was observed. In contrast, the total male centennial population presented an average of 23.86%. With the descriptive values found, a statistically significant difference in favor of the female sex (t=5.381; df=36; p<0.05) and centennial female sex was evidenced (t=59.271; df=36; p<0.05).

At the provincial level, the normalized total female population showed an average of 51.02%. Similarly, the relativized total male population presented an average of 48.98%. The total centennial female population had an average of 75.47%. On the other hand, the male centennial population showed an average of 24.53%. With these data obtained from the descriptive analysis, a statistically positive difference was established in favor of the female sex (t=11.928; df=102; p<0.05) and centennial female sex (t=90.119; df=102; p<0.05).

**Figure 3:**
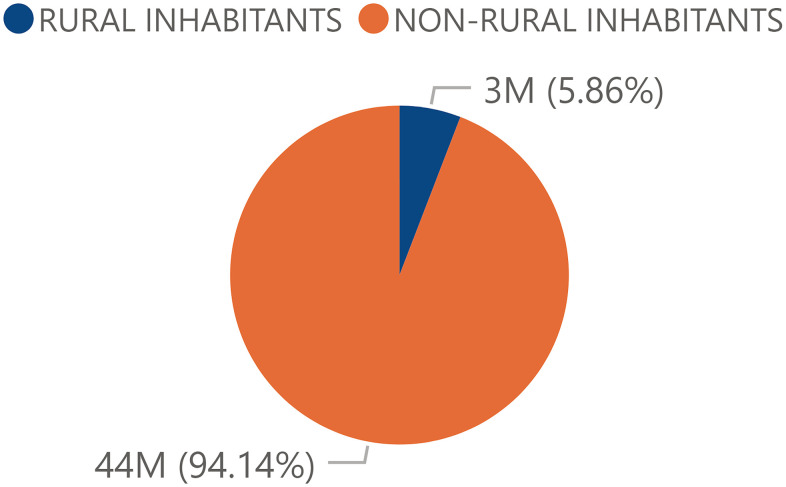
**Percentage of the total population from rural and urban areas.** Percentage of the total Spanish population by rurality in 2017 obtained from the INE.

**Figure 4:**
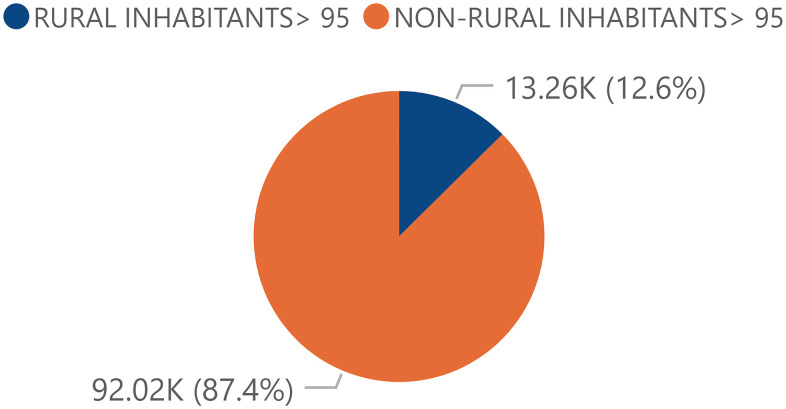
**Percentage of the total centennial population from rural and urban areas.** Percentage of the total Spanish centennial population by rurality in 2017 obtained from the INE.

The total Spanish population had a significant tendency of distribution towards the urban or non-rural areas. Thus, a relativized total non-rural population of 94.14% and a rural population of 5.86% was observed (Figure 3). The centennial population followed a similar pattern to that of the total population: there was a higher proportion of individuals in urban areas (87.4%) than in rural areas (12.60%) (Figure 4).

A distribution of the total population was described with a tendency towards urban centers (non-rural) being very significant in the Autonomous communities of Andalucía, Asturias, Islas Baleares, Islas Canarias, Cataluña, Comunidad Valenciana, Madrid, Murcia and País Vasco which presented a relativized non-rural population (Supplementary Table 4). On the contrary, Castilla y León presented the highest autonomous rural population in Spain (Supplementary Table 4). When analyzing the total centennial population, the pattern was similar: there was a greater trend towards urban areas. The territories of the Islas Baleares, Islas Canarias, Madrid and Murcia were those with the highest total centennial population within the non-rural areas (Supplementary Table 5). On the contrary, Castilla y León and Extremadura were the regions with a lower tendency towards urban areas (Supplementary Table 5).

At the provincial level of the total Spanish population, the distribution followed the same trend: most of the territories had a larger urban (non-rural) population, well above the rural population. The provinces that presented a greater proportion of urban population and consequently a greater percentage difference between it and the rural population were Cádiz, Córdoba, Huelva, Malaga, Sevilla, Asturias, Islas Baleares, Las Palmas, Santa Cruz de Tenerife, Barcelona, Alicante, Valencia, A Coruña, Pontevedra, Madrid, Murcia, Guipúzcoa and Vizcaya. On the contrary, Teruel and Zamora were the provinces with a higher proportion of rural population (Supplementary Table 6). In the centenary Spanish population, the distribution followed the same urban (non-rural) trend in most territories, being the provinces of Cádiz, Málaga, Sevilla, Islas Baleares, Las Palmas, Santa Cruz de Tenerife, Barcelona, Alicante, A Coruña, Pontevedra, Madrid, Murcia, Guipúzcoa and Vizcaya those that presented a greater proportion of non-rural centennial population (Supplementary Table 7). However, there were territories that adopt a totally opposite trend, and presented an inclination towards rural areas. These territories were the provinces of Teruel, Cuenca, Ávila, Segovia and Zamora (Supplementary Table 7).

When the inferential analysis was applied, regarding non-rural areas at the regional level, the female population had an average with a value of 50.97% and male population had an average of 49.03%. The female centennial population an average of 76.68% was observed. In contrast, the male centennial population had an average with a value of 23.32%. Once these descriptive data were obtained, a statistically significant difference was shown in favor of the female sex (t=6.654; df=36; p<0.05) and centennial female sex (t=60.500; df=36; p<0.05). In relation to the rural areas, it was decided to exclude Ceuta and Melilla because they had a rural population equal to 0. The female population showed an average of 48.12% while male population had an average of 51.88%. With these descriptive data, a statistically positive difference was described in favor of the male sex (t=-10.729; df=32; p<0.05). The centennial female population had a mean value of 72.42% as long as the male centennial population had an average of 27.58%. With these values obtained from the descriptive analysis, a statistically significant difference was described in favor of the centennial female sex (t=23.491; df=32; p<0.05).

Regarding non-rural areas at the provincial level, the female population showed an average of 51.02%. On the other hand, in the male population an average of 48.98% was observed. The centennial female population presented an average with a value of 76.42% and the male population of 23.58%. With values obtained from the descriptive data, a statistically positive difference was observed in favor of the female sex (t=11.928; df=102; p<0.05) and centennial female sex (t=95.730; df=102; p<0.05). Regarding rural areas, the female population had an average with a value of 48.11%. In contrast, the male population had an average of 51.89%. With these descriptive data, a statistically significant difference was observed in favor of the male sex (t=-14.004; df=98; p<0.05). The female centennial population showed an average of 72.05%. On the opposite, the male centennial population had an average of 27.95%. Once these descriptive data were obtained, a statistically significant difference was observed in favor of the centennial female sex (t=40.941; df=98; p<0.05).

In order to contrast a possible interaction between the two factors, sex and rurality, with respect to the dependent variable inhabitants (population > 95 years/1000inhabitants), it was carried out a two-factor ANOVA test (F=32.033; df=1; p<0.05), resulting in a significant interaction between both variables. We found that the centennial population was differently distributed depending on the rurality of the national territory. Rurality is a determining factor in the long-lived Spanish population of both sexes. Additionally, we studied if rurality has an effect on long-living in the population of the same sex (i.e. rural men vs non-rural men, rural women vs non-rural women): we found that rurality significantly (p<0.05) favors the longevity of the population, independently of the sex in the Spanish state.

**Figure 5:**
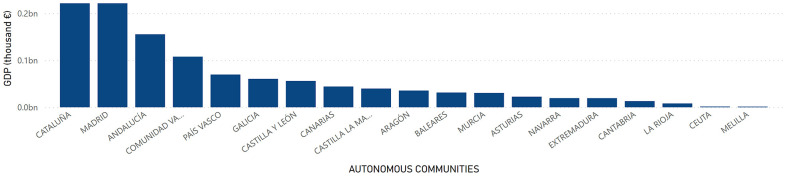
**GDP by Autonomous Communities.** Quantity (in thousand €) of GDP in the Autonomous Communities in Spain.

**Figure 6:**
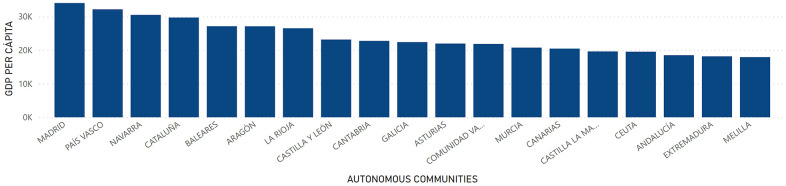
**GDP per Capita by Autonomous Communities.** Quantity (in thousand €) of GDP per Capita in the Autonomous Communities in Spain.

The socioeconomic factor had been established as a dependent variable in the present study. It was assumed that changes in this factor could generate changes in the longevity of people. In this way, GDP and GDP per Capita were collected as markers of the socioeconomic factor, and the following distribution was found at the regional level: a higher GDP was appreciated in Andalucía, Cataluña and Madrid with a total of 155,462,810; 221,437,086 and 221,432,620 thousand euros, respectively. In contrast, Melilla with 1,520,977 thousand euros, Ceuta with 1,660,550 thousand euros and La Rioja with 8,287,052 thousand euros presented the lowest GDP in Spain (Figure 5). Regarding GDP per Capita, the territories of Madrid, País Vasco and Navarra, with figures of 34,041; 32,167 and 30,508 euros, respectively, presented the highest figures. On the contrary, the Autonomous Communities of Melilla, Extremadura and Andalucía with a total of 17,934; 18,170 and 18,501 euros, respectively, were the regions with the lowest figures (Figure 6).

**Figure 7:**
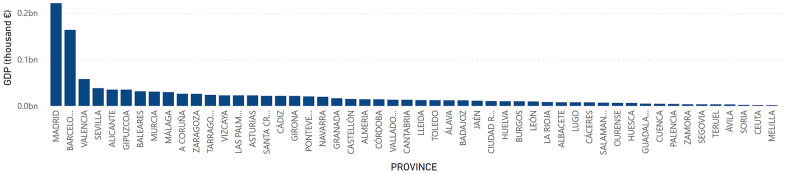
**GDP by provinces.** Quantity (in thousand €) of GDP in the provinces in Spain.

**Figure 8:**
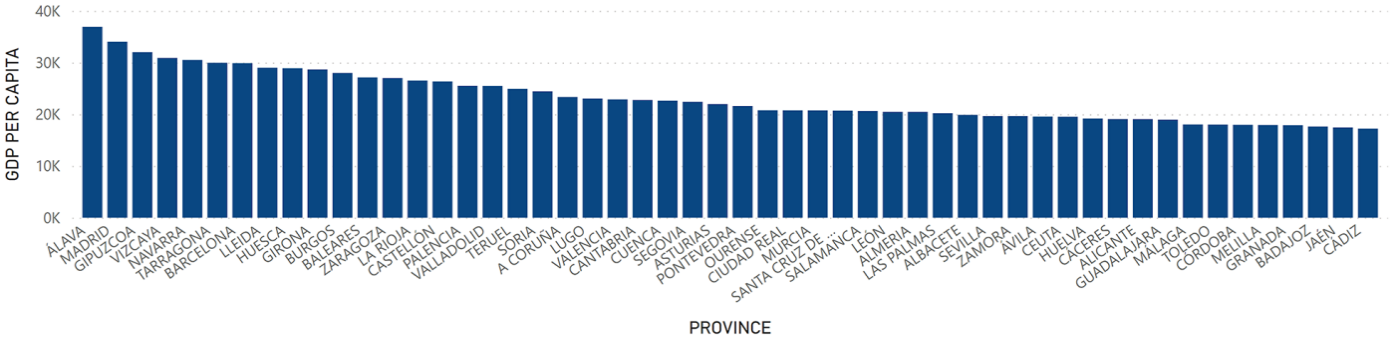
**GDP per Capita by provinces.** Quantity (in thousand €) of GDP per Capita in the provinces in Spain.

At the provincial level, GDP had a higher value in the territories of Madrid with a total of 221,432,620 thousand euros, Barcelona with 163,829,876 thousand euros and Valencia with 57,719,399 thousand euros. On the other hand, the provinces of Melilla with a GDP of 1,520,977 thousand euros; Ceuta with 1,660,550 thousand euros; and Soria with 2,192,878 thousand euros were those with the lowest levels of GDP in Spain (Figure 7). Regarding GDP per Capita, Álava, Madrid and Vizcaya presented the highest figure with a value of 36,921; 34,041 and 32,015 euros, respectively. On the other hand, Cádiz, Jaén and Badajoz presented the lowest figures with 17,231; 17,465 and 17,637 euros, respectively (Figure 8).

To find and estimate the impact of GDP per Capita on longevity by rural areas, the Pearson correlation test was used applying the statistical program R. The motivation to separate the analysis of the centennial population by rural areas and by sex was justified after showing statistically significant differences between the population distribution according to sex.

At the regional level, GDP per Capita did not show statistically significant correlations with the non-rural centennial population (correlation=0.444; t=2.041; df=17; p=0.057). Similarly, the correlation between GDP per Capita and the rural centennial population was not statistically significant neither (correlation=-0.042; t=-0.174; df=17; p=0.863). GDP per Capita showed a statistically significant correlation with the non-rural female centennial population (correlation=0.475; t=2.227; df=17; p=0.039). In contrast, the homologous correlation between GDP per Capita and the female rural centennial population was not statistically significant (correlation=-0.030; t=-0.127; df=17; p=0.902). GDP per Capita did not show a statistically significant correlation with the non-rural male centennial population (correlation=0.292; t=1.258; df=17; p=0.225). The correlation between GDP per Capita and the male rural centennial population was not statistically significant either (correlation=-0.069; t=-0.288; df=17; p=0.776).

Regarding the provinces, the correlation between GDP per Capita and the non-rural centennial population (over 95 years old) did not reach statistical significance at 0.05 neither (correlation=0.287; t=2.120; df=50; p=0.389). Similarly, the correlation observed between GDP per Capita and the rural centennial population was not statistically significant neither (correlation=-0.073; t= -0.520; df=50; p=0.605). In relation to the provinces, the correlation between GDP per Capita and the non-rural female centennial population was statistically significant (correlation=0.334; t=2.508; df=50; p=0.015) while the correlation observed between GDP per Capita and the female rural centennial population was not (correlation=-0.067; t=-0.478; df=50; p=0.634). In relation to centennial men population, there were not statistically significant correlations between GDP per Capita and the non-rural men (correlation=0.110; t=0.784; df=50; p=0.436) and neither between GDP per Capita and the rural centennial men (correlation=-0.085; t=-0.609; df=50; p=0.545).

With respect to the interaction of rurality and sex, as mentioned above, the ANOVA analysis with two factors (rurality and sex) showed the existence of statistically significant differences regarding the interaction of the rurality and sex variables (F=32.022; df=1; p<0.05). Finally, in view of the interest in knowing the interaction between GDP, an interval variable, and the rest of the variables, the provincial GDP per Capita variable was divided into 6 intervals using the Sturges technique [24]. This allowed the data to be entered into the SPSS program in which the multivariate ANOVA was performed, used both for the study of the previous interaction of the sex and rurality factors and in the following: interaction between GDP with the rest of variables (sex and rurality). The ANOVA analysis with two factors (GDP per Capita and sex) did not show statistically significant differences regarding the interaction of the variables GDP and sex (F= 1.500; df=5; p>0.05). The two factors ANOVA analysis (GDP per Capita and rurality) did neither show statistically significant differences regarding the interaction of the variables GDP and rurality (F=2.229; df=5; p>0.05). Moreover, the three factors ANOVA analysis including GDP per Capita, sex and rurality did neither show statistically significant differences regarding their interaction (F=0.769; df=5; p>0.05). Then, there were only statistically significant differences in favor of the female centennial population and non-rural areas taking into account the GDP, both at the regional and at the provincial levels (Table 1).

**Table 1:** Correlations between GDP with the variables sex and rurality on the centennial population.

		**Women**			**Men**	
		**Non-rural**	**Rural**		**Non-rural**	**Rural**
**GDP**	**Autonomous Communities**	p=0.03972*	p=0.9002		p=0.2252	p=0.7761
	**Provinces**	p=0.01542*	p=0.6346		p=0.4364	p=0.5452

Correlation of P-value taking into account the GDP, showing significant differences in non-rural centennial women in the Autonomous Communities and Provinces.

## DISCUSSION

**Figure 9:**
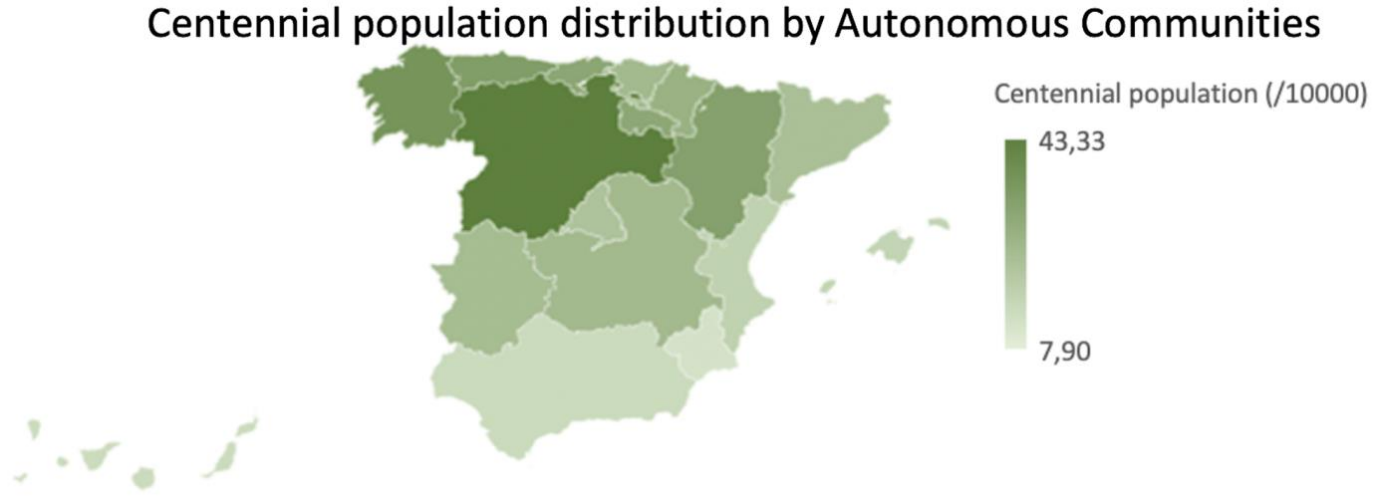
**Centennial population distribution by Autonomous Communities.** Normalized centennial population (per 10.000 inhabitants) by Autonomous Communities.

The aim of this study was to evaluate the possible impact of sex, socioeconomic factors, specifically GDP per Capita, and rurality on the prevalence of centennial population (> 95 years) in the Spanish society, through the 2017 continuous population census. First of all, it must be considered that the Spanish territory showed a heterogeneous distribution regarding centennial society (Figure 9). Regarding the sex variable in the total population, a significant difference was seen in favor of the female sex (Figure 1) and this trend was even exacerbated in the centennial population (Figure 2): there were significantly more centennial women than men in the Spanish society [22, 23]. It is understood that there could be gender factors that might determine this trend towards a larger centennial female population in Spain, but possibly not only genetic but also sociocultural aspects, even if they are aspects that remain open without an answer in the literature although it is a universal fact in all countries [25, 26]. For instance, a Chinese study corroborates that women tend to significantly live longer than men but experience worse health compared to men [13].

**Figure 10:**
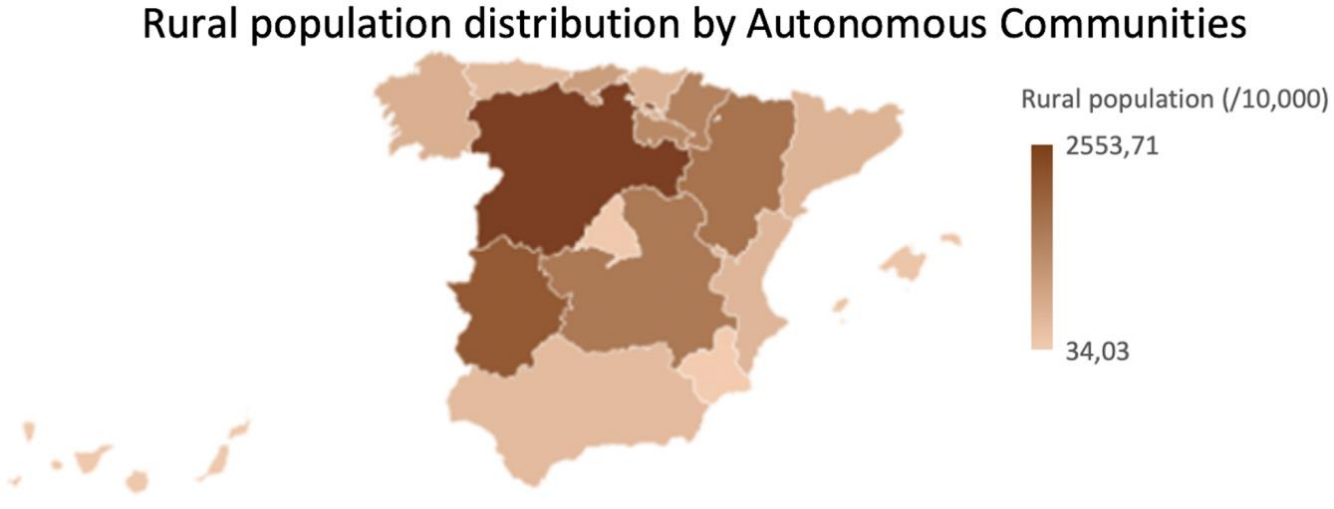
**Rural population distribution by Autonomous Communities.** Normalized rural population (per 10.000 inhabitants) by Autonomous Communities.

In the total population, when the comparison was done by rural areas, it was observed that the weight of the female population was greater in urban areas and the male population was greater in rural areas. However, this distribution varied radically when the centennial population was analyzed as the findings showed a significant higher female centennial population compared to centennial men in both rural and urban areas. The distribution of the centennial population, both female and male, was higher in the rural areas, and this had a similarity between the distribution of centennial population in the Autonomous Communities (Figure 9) with the distribution of rural population by Autonomous Communities (Figure 10). Some authors support that living in rural areas can be a determining factor for people's survival, since rural centenarians enjoyed better health status (measured by ADL ability and physical performance tests) [13] and exhibited greater cardiovascular disease age-adjusted mortality rates [27] while the urban centenarians report to have better life satisfaction [28]. These results let us think that living in rural areas may force people to perform daily activities for a longer time than people from urban areas [13]. In addition to this, centenarians from rural areas still work in the fields, that may help to maintain the physical capacity [13]. Therefore, older people living in rural areas enjoys better quality of life than living in urban areas [29]. Contrary to this, a study performed in Japan regarding rural found that elderly ruralites had a higher risk than urban counterparts of being ADL or Instrumental Activities of Daily Living (IADL) disabled but had a lower chance of depressive symptoms [30].

**Figure 11:**
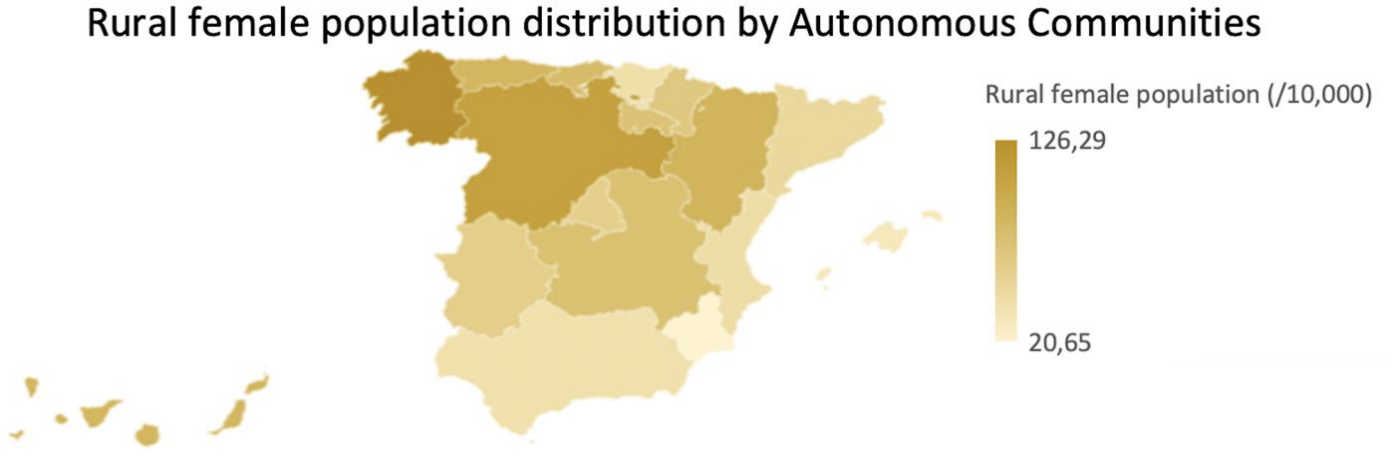
**Rural female population distribution map by Autonomous Communities.** Normalized rural female population (per 10.000 inhabitants) by Autonomous Communities.

Through the analysis with the two-factor ANOVA, a significant interrelation of the three variables (sex, rurality and longevity) was established. In fact, the female centennial population from rural territories is older (Figure 11). Moreover, it has been shown that the proportion of elderly rural Russian women was significantly higher than urban women [31]. In addition, the proportion of long-lived people who cannot service themselves was twice as large than countryside; this could be explained that rural residents remained more active in self-service than townspeople and this last group (urban people) had at least one and a half times more chronic diseases than rural residences [31]. Even if older women live longer, they were less functional in IADL and had greater disability than men [29].

**Figure 12:**
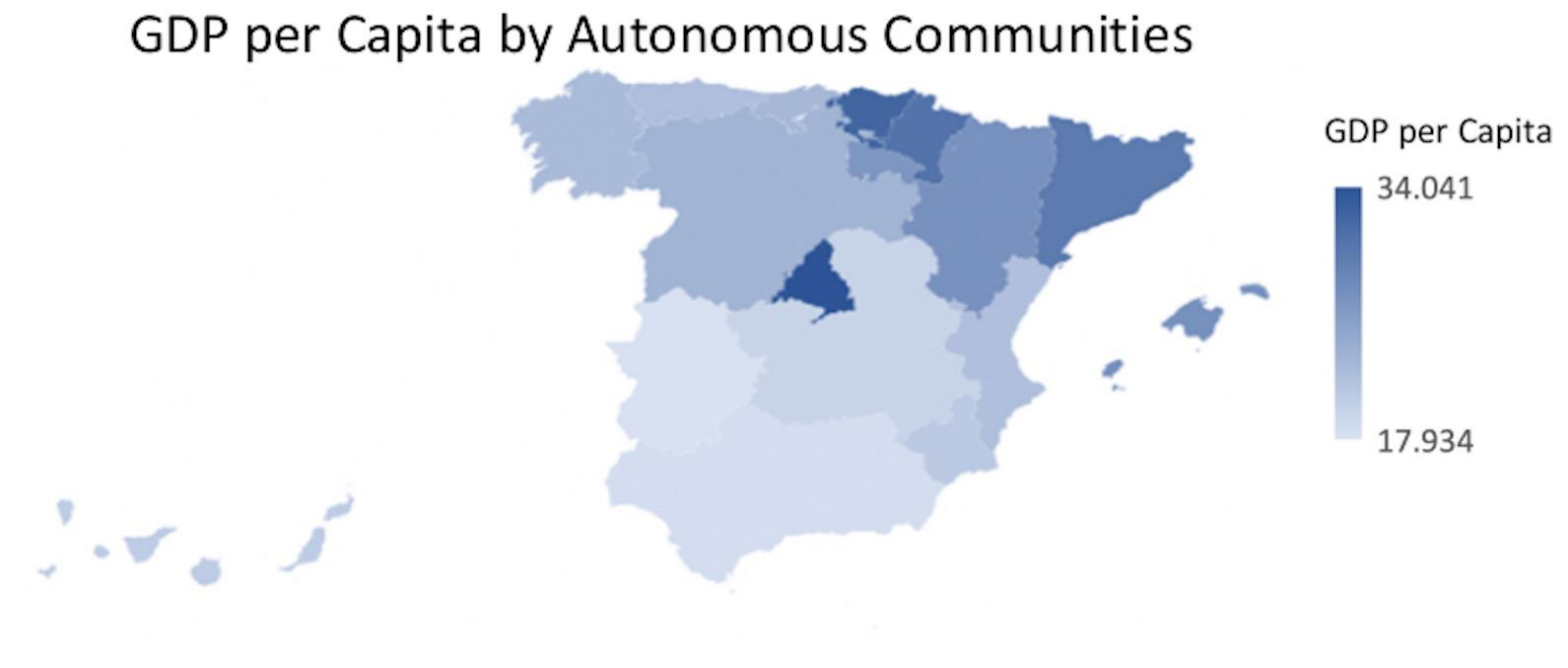
**GDP per Capita distribution map by Autonomous Communities.** Normalized proportion of GDP per Capita by Autonomous Communities.

It was observed that the areas with higher GDP per Capita (Figure 12) did not match with the areas with more centennial population (Figure 9).

After the inferential Pearson analysis, a null correlation was observed between the GDP per Capita and the rurality variable on the total centennial population, both at the regional and provincial levels. When introducing the gender variable, a null correlation was also observed with the centennial male population, both in rural and non-rural areas. However, a statistically significant relationship was observed between the economic factor, non-rural areas and the female centennial population, both at the regional level and at the provincial level. This would indicate that, in urban areas, the female centennial population had a higher GDP per Capita. On the contrary, in rural areas, neither the female nor the male population showed a correlation with GDP. This could be due to the fact that, upon reaching a certain age, the population migrate to urban centers where they can find better benefits and better services adapted to the needs of their advanced age [30]. Contrary to this, the data from a research performed in China showed that centennial men had a higher socioeconomic status than women and most female centenarians with no schooling were living in the rural areas [13].

No significant correlation was observed when analyzing the interactions between the three variables (sex, age and GDP). The statistical analysis revealed that GDP alone was not a determining factor and it was not decisive in increasing people's longevity (F=4.725; df=5; p>0.05). In this sense, authors supported that the economy of a territory weighs heavily on the survival of the centennial population, both due to the level of family income [4] and the greater degree of urban planning that a region showed [32]. This latter would support the interpretation of the results of the present study since, although there were no statistically significant differences between GDP and rurality in the Spanish centennial population (F=2.229; df=5; p>0.05), there was a significant correlation of GDP with centennial women who live in urban areas. Therefore, the degree of urban planning could participate as a survival variable, representing greater accessibility to better health services [32]. Having higher values of GDP per capita and lower levels of infant mortality confirms an increase in longevity [33].

The influence of the variables under study (sex, rurality and GDP) independently indicated that: i) sex influences in longevity as the female centennial population is higher compared to the male centennial population; ii) rurality was a determining factor in the long-lived population in Spain as rural areas had a significantly higher proportion of the centennial population; iii) in general, the socioeconomic factor (GDP per Capita) as an independent variable was not a determinant in longevity of the Spanish population; but iv) there were statistically significant differences taking into account GDP with the variables sex and rurality on the centennial population, specifically in favor of the female centennial population and non-rural areas, at the regional and at the provincial level.

### Future research

It should be noted that the centennial population analyzed in this research lived its adolescence and youth in the extremely poor decades of the 20s and 30s of the last century, and that they lived famines and the hardships of the Spanish Civil War (1936-1939). Taking into account that the socioeconomic factor (and poor nutrition) strongly influences the pubertal cycle of an individual [34], it would be advisable for future research to analyze this strong period of poverty (and famine) as a disruptive element of the correlation between GDP and longevity in this kind of studies.

It is of great interest that other types of variables could be included, such as, for example, pollution levels in Spain: the number of registrations by territories, population transfer, existing public transport, etc. Also highlight the family factor, that is, evaluate whether the centennial population lives alone or with someone (either in residences or at home with at least one relative) as a variable of interest to determine correlations with family and social support, and / or individual happiness [4, 6, 7]. In addition to this, it would be interesting to include the healthcare accessibility in rural and urban areas as it is not a simple concept and in a previous study residing in rural areas was not clearly related to diagnostic delays, greater morbidity or mortality [32]. Furthermore, it will be fascinating to explore the conditions of the centenarian offspring’s (quality of life, how old are they, etc.), as it has been showed that centenarian offspring showed an intermediate immunophenotype between age-matched and younger controls [35].

It is noteworthy that the present results open the door to carry out more in-depth and extensive analyses, introducing other dependent variables that may influence longevity in order to provide new evidence for this phenomenon such as ZIP code within each province, family situation, cultural level, or environmental contamination. It is also important that future research will study the situation of women in their 90s and 100s in greater depth to improve their quality of life especially when they are survivors, and it is striking that they survive even with lower GDP.

### Limitations

The main difficulty in this study was the lack of data referring to the population over 100 years old in some autonomous regions, so it was chosen to include the entire group of people over 95 years old in the study. Likewise, although it would have been desired to use data from 2019, it was impossible since some Autonomous Communities and provinces did not have these data so for uniformity criteria the municipal register of 2017 was used as it was the closest that included the data referred to GDP per Capita. In addition, it was intended to carry out the study by municipalities; unfortunately, it was not possible to obtain GDP data at the municipal level. Not having municipal information on the address and/or family situation of the centennial population has limited the analysis and led to the exclusion of the socio-family factor from the study.

All municipalities with less than 2,000 inhabitants were considered as rural areas regarding the information provided by the National Institute of Statistics (INE) and the definition of rurality, which from the sociocultural point of view moves away from the rural reality, which could imply an interpretation bias.

## MATERIALS AND METHODS

### Study design, setting, and patients

For the present study, a sample made up of the 46,572,132 inhabitants in Spain (Spanish population and foreign population officially residing in the country) and the socioeconomic factor were selected from the INE in 2017. The variables for the study were population, age, sex, socioeconomic factor and rurality. Taking into account that GDP breaks down the community’s economic output, for the purpose of analyzing the socioeconomic factor, the parameters GDP and GDP per Capita were considered by Autonomous Communities and provinces.

Within the total Spanish population, 23,739,271 was the total female population of both Spaniards and foreign women and 22,832,861 was the total male population of Spaniards and foreign males. Then, from this sample, 105,279 individuals corresponded to Spanish people that were 95 years old or older. This centennial population (both Spaniards and foreigners) was subdivided by sex being 80,783 the total female centennial population and 24,496 the total male centennial population.

In this study, rurality was understood as municipalities with a population equal to or less than 2,000 inhabitants (data obtained from INE in 2017). The sample included 2,728,017 individuals as “rural population”, with a total of 1,310,477 women and 1,417,540 men. Subsequently, the “centennial rural population” was of 13,262 people, in which 9,577 were women and 3,685 were men (Table 2).

**Table 2:** Rural population and centennial rural population.

	**Absolute values**	**Percentage**
**Population**	2,728,017	100%
**Women**	1,310,477	48.04%
**Men**	1,417,540	51.96%
**Population > 95 years old**	13,262	100%
**Women > 95 years old**	9,577	72.21%
**Men > 95 years old**	3,685	27.79%

Absolute and percentage figures of total society by sex obtained from the INE (2017).

### Statistical analysis

Three analyses were carried out. The first analysis was a descriptive study in order to know the average and distributive behavior (central tendency data) of the socioeconomic and demographic data referring to total and centennial population by sex, by rural areas (rural and non-rural) and by GDP in the different Spanish Autonomous Communities and provinces. The second analysis was the inferential analysis to determine a priori statistically significant differences (independent T-Student test and ANOVA) regarding the distribution of total and centennial population by sex in Autonomous Communities and provinces; and by rural areas and sex in Autonomous Communities and provinces. The third and last analysis was the inferential analysis (Pearson's correlation and ANOVA of two and three factors) to demonstrate the possible impact of socioeconomic factors (GDP) on the prevalence between the centennial population by rural areas, between the female and male centennial population by rural areas, in Autonomous Communities and provinces and the GDP; and the interaction of rurality and sex; of GDP and sex; of GDP and rurality; and of rurality, sex and GDP on the centennial population.

The results obtained were carried out with the analysis of the variance of repeated measures with a confidence level of 95% and a significance level of α = 0.05.

## Supplementary Material

Supplementary Tables
