# Supplementary material for: Sex, rurality and socioeconomical status in Spanish centennial population (2017)
Source: Aging (Albany NY). 2021 Sep 26;13(18):22059–77. doi: 10.18632/aging.203563 (PMC8507300; doi:10.18632/aging.203563)
Supplement: Supplementary Tables [file aging-13-203563-s001.pdf]

SUPPLEMENTARY TABLES

Supplementary Table 1. Total population by sex in 2017.

Autonomous community	% Women	% Men	Autonomous community	% Women	% Men
**ESPAÑA**	50,97	49,03	**COMUNIDAD VALENCIANA**	50,70	49,30
**ANDALUCÍA**	50,67	49,33	**EXTREMADURA**	50,45	49,55
**ARAGÓN**	50,65	49,35	**GALICIA**	51,84	48,16
**ASTURIAS**	52,28	47,72	**LA RIOJA**	50,69	49,31
**BALEARES**	50,28	49,72	**MADRID**	52,12	47,88
**CANARIAS**	50,42	49,58	**MURCIA**	49,93	50,07
**CANTABRIA**	51,44	48,56	**NAVARRA**	50,46	49,54
**CASTILLA LA MANCHA**	49,95	50,05	**PAÍS VASCO**	51,38	48,62
**CASTILLA Y LEÓN**	50,73	49,27	**CEUTA**	49,35	50,65
**CATALUÑA**	50,90	49,10	**MELILLA**	49,34	50,66

Percentage of the total Spanish population by sex and by Autonomous Communities obtained from the INE.

Supplementary Table 2. Total centennial population by sex in 2017.

Autonomous community	% Women	% Men	Autonomous community	% Women	% Men
**ESPAÑA**	76,73	23,27	**COMUNIDAD VALENCIANA**	75,74	24,26
**ANDALUCÍA**	74,94	25,06	**EXTREMADURA**	74,02	25,98
**ARAGÓN**	77,34	22,66	**GALICIA**	75,72	24,28
**ASTURIAS**	78,31	21,69	**LA RIOJA**	76,17	23,83
**BALEARES**	76,99	23,01	**MADRID**	79,33	20,67
**CANARIAS**	72,26	27,74	**MURCIA**	72,91	27,09
**CANTABRIA**	80,07	19,93	**NAVARRA**	77,46	22,54
**CASTILLA LA MANCHA**	73,11	26,89	**PAÍS VASCO**	81,32	18,68
**CASTILLA Y LEÓN**	75,31	24,69	**CEUTA**	71,01	28,99
**CATALUÑA**	78,11	21,89	**MELILLA**	76,47	23,53

Percentage of the total Spanish centennial population by sex and by Autonomous Communities obtained from the INE.

Supplementary Table 3. Total centennial population by sex in 2017.

Province	% Women	% Men	Province	% Women	% Men
ESPAÑA	76,73	23,27	SEGOVIA	76,95	23,05
ALMERIA	70,62	29,38	SORIA	74,48	25,52
CÁDIZ	76,78	23,22	VALLADOLID	77,98	22,02
CÓRDOBA	75,35	24,65	ZAMORA	69,71	30,29
GRANADA	74,38	25,62	BARCELONA	79,19	20,81
HUELVA	76,77	23,23	GIRONA	76,17	23,83
JAÉN	73,16	26,84	LLEIDA	74,86	25,14
MÁLAGA	72,39	27,61	TARRAGONA	74,49	25,51
SEVILLA	77,9	22,1	ALICANTE	75,06	24,94
HUESCA	73,37	26,63	CASTELLÓN	74,92	25,08
TERUEL	73,64	26,36	VALENCIA	76,4	23,6
ZARAGOZA	79,35	20,65	BADAJOZ	76,75	23,25
ASTURIAS	78,31	21,69	CÁCERES	71,29	28,71
BALEARES	76,99	23,01	A CORUÑA	76,91	23,09
LAS PALMAS	73,01	26,99	LUGO	72,25	27,75
SANTA CRUZ DE TENERIFE	71,65	28,35	OURENSE	75,23	24,77
CANTABRIA	80,07	19,93	PONTEVEDRA	76,68	23,32
ALBACETE	71,36	28,64	LA RIOJA	76,17	23,83
CIUDAD REAL	75,41	24,59	MADRID	79,33	20,67
CUENCA	72,13	27,87	MURCIA	72,91	27,09
GUADALAJARA	72,86	27,14	NAVARRA	77,46	22,54
TOLEDO	72,9	27,1	ÁLAVA	80,39	19,61
ÁVILA	75,61	24,39	GIPUZCOA	81,88	18,12
BURGOS	77,21	22,79	VIZCAYA	81,21	18,79
LEÓN	73,95	26,05	CEUTA	71,01	28,99
PALENCIA	79,14	20,86	MELILLA	76,47	23,53
SALAMANCA	74,11	25,89			

Percentage of the total Spanish centennial population by sex and by provinces obtained from the INE.

Supplementary Table 4. Total population by rurality in 2017.

Autonomous community	% Non-rural inhabitants	% Rural inhabitants	Autonomous community	% Non-rural inhabitants	% Rural inhabitants
ESPAÑA	94,14	5,86	CATALUÑA	95,46	4,54
ANDALUCÍA	96,73	3,27	COMUNIDAD VALENCIANA	95,86	4,14
ARAGÓN	83,93	16,07	EXTREMADURA	79,59	20,41
ASTURIAS	96,28	3,72	GALICIA	94,71	5,29
BALEARES	98,64	1,36	LA RIOJA	88,00	12,00
CANARIAS	99,19	0,81	MADRID	99,17	0,83
CANTABRIA	91,55	8,45	MURCIA	99,66	0,34
CASTILLA LA MANCHA	85,10	14,90	NAVARRA	86,75	13,25
CASTILLA Y LEÓN	74,46	25,54	PAÍS VASCO	95,07	4,93

Percentage of the total Spanish population based on rural and non-rural areas and by Autonomous Communities obtained from the INE.

Supplementary Table 5. Total centennial population by rurality in 2017.

Autonomous community	% Non-rural inhabitants >95	% Rural inhabitants >95	Autonomous community	% Non-rural inhabitants >95	% Rural inhabitants >95
**ESPAÑA**	87,40	12,60	**CATALUÑA**	93,41	6,59
**ANDALUCÍA**	94,23	5,77	**COMUNIDAD VALENCIANA**	92,92	7,08
**ARAGÓN**	72,08	27,92	**EXTREMADURA**	64,78	35,22
**ASTURIAS**	93,89	6,11	**GALICIA**	87,39	12,61
**BALEARES**	98,11	1,89	**LA RIOJA**	82,28	17,72
**CANARIAS**	96,87	3,13	**MADRID**	98,68	1,32
**CANTABRIA**	87,21	12,79	**MURCIA**	99,50	0,50
**CASTILLA LA MANCHA**	70,25	29,75	**NAVARRA**	80,93	19,07
**CASTILLA Y LEÓN**	58,30	41,70	**PAÍS VASCO**	94,84	5,16

Percentage of the total Spanish centennial population according to rural and non-rural areas and Autonomous Communities obtained from the INE.

Supplementary Table 6. Total population by rurality in 2017.

Province	% Non-rural inhabitants	% Rural inhabitants	Province	% Non-rural inhabitants	% Rural inhabitants
**ESPAÑA**	94,14	5,86	**SALAMANCA**	71,04	28,96
**ALMERIA**	94,70	5,30	**SEGOVIA**	64,04	35,96
**CÁDIZ**	99,48	0,52	**SORIA**	71,62	28,38
**CÓRDOBA**	97,01	2,99	**VALLADOLID**	88,71	11,29
**GRANADA**	91,00	9,00	**ZAMORA**	52,17	47,83
**HUELVA**	95,18	4,82	**BARCELONA**	98,71	1,29
**JAÉN**	93,09	6,91	**GIRONA**	87,39	12,61
**MÁLAGA**	97,64	2,36	**LLEIDA**	76,49	23,51
**SEVILLA**	99,17	0,83	**TARRAGONA**	90,60	9,40
**HUESCA**	68,52	31,48	**ALICANTE**	97,57	2,43
**TERUEL**	59,81	40,19	**CASTELLÓN**	90,52	9,48
**ZARAGOZA**	90,91	9,09	**VALENCIA**	95,84	4,16
**ASTURIAS**	96,28	3,72	**BADAJOZ**	86,46	13,54
**BALEARES**	98,64	1,36	**CÁCERES**	67,91	32,09
**LAS PALMAS**	99,66	0,34	**A CORUÑA**	98,04	1,96
**SANTA CRUZ DE TENERIFE**	98,67	1,33	**LUGO**	89,53	10,47
**CANTABRIA**	91,55	8,45	**OURENSE**	73,98	26,02
**ALBACETE**	87,67	12,33	**PONTEVEDRA**	99,43	0,57
**CIUDAD REAL**	89,86	10,14	**LA RIOJA**	88,00	12,00
**CUENCA**	63,32	36,68	**MADRID**	99,17	0,83
**GUADALAJARA**	82,40	17,60	**MURCIA**	99,66	0,34
**TOLEDO**	87,46	12,54	**NAVARRA**	86,75	13,25
**ÁVILA**	64,78	35,22	**ÁLAVA**	90,40	9,60
**BURGOS**	75,75	24,25	**GIPUZCOA**	96,00	4,00
**LEÓN**	76,27	23,73	**VIZCAYA**	95,82	4,18
**PALENCIA**	73,12	26,88			

Percentage of the total Spanish population based on rural and non-rural areas and by provinces obtained from the INE.

Supplementary Table 7. Total centennial population by rurality in 2017.

Province	% Non-rural inhabitants >95	% Rural inhabitants >95	Province	% Non-rural inhabitants >95	% Rural inhabitants >95
**ESPAÑA**	87,40	12,60	**SALAMANCA**	57,18	42,82
**ALMERIA**	87,15	12,85	**SEGOVIA**	48,86	51,14
**CÁDIZ**	99,17	0,83	**SORIA**	57,84	42,16
**CÓRDOBA**	94,78	5,22	**VALLADOLID**	77,28	22,72
**GRANADA**	88,57	11,43	**ZAMORA**	31,21	68,79
**HUELVA**	84,34	15,66	**BARCELONA**	98,32	1,68
**JAÉN**	89,37	10,63	**GIRONA**	84,74	15,26
**MÁLAGA**	97,36	2,64	**LLEIDA**	66,89	33,11
**SEVILLA**	98,61	1,39	**TARRAGONA**	84,85	15,15
**HUESCA**	56,52	43,48	**ALICANTE**	96,40	3,60
**TERUEL**	44,11	55,89	**CASTELLÓN**	79,75	20,25
**ZARAGOZA**	82,71	17,29	**VALENCIA**	94,08	5,92
**ASTURIAS**	93,89	6,11	**BADAJOZ**	77,68	22,32
**BALEARES**	98,11	1,89	**CÁCERES**	51,89	48,11
**LAS PALMAS**	98,68	1,32	**A CORUÑA**	96,56	3,44
**SANTA CRUZ DE TENERIFE**	95,38	4,62	**LUGO**	82,85	17,15
**CANTABRIA**	87,21	12,79	**OURENSE**	56,63	43,37
**ALBACETE**	78,16	21,84	**PONTEVEDRA**	97,91	2,09
**CIUDAD REAL**	83,15	16,85	**LA RIOJA**	82,28	17,72
**CUENCA**	46,94	53,06	**MADRID**	98,68	1,32
**GUADALAJARA**	55,88	44,12	**MURCIA**	99,50	0,50
**TOLEDO**	73,20	26,80	**NAVARRA**	80,93	19,07
**ÁVILA**	43,90	56,10	**ÁLAVA**	86,84	13,16
**BURGOS**	64,68	35,32	**GIPUZCOA**	97,37	2,63
**LEÓN**	62,22	37,78	**VIZCAYA**	95,39	4,61
**PALENCIA**	55,86	44,14			

Percentage of the total Spanish centennial population according to rural and non-rural areas and provinces obtained from the INE.
